# 1-{(*E*)-[3-(1*H*-Imidazol-1-yl)-1-phenyl­propyl­idene]amino}-3-(2-methyl­phen­yl)urea

**DOI:** 10.1107/S1600536812022659

**Published:** 2012-05-23

**Authors:** Mohamed I. Attia, Mohamed N. Aboul-Enein, Nasser R. El-Brollosy, Seik Weng Ng, Edward R. T. Tiekink

**Affiliations:** a Department of Pharmaceutical Chemistry, College of Pharmacy, King Saud University, Riyadh 11451, Saudi Arabia; b Medicinal and Pharmaceutical Chemistry Department, Pharmaceutical and Drug Industries Research Division, National Research Centre, 12622 Dokki, Giza, Egypt; c Department of Chemistry, University of Malaya, 50603 Kuala Lumpur, Malaysia; d Chemistry Department, Faculty of Science, King Abdulaziz University, PO Box 80203 Jeddah, Saudi Arabia

## Abstract

In the title compound, C_20_H_21_N_5_O, the conformation about the imine bond [1.289 (3) Å] is *E*. Overall, the mol­ecule is disk-shaped with the imidazole ring located above the remainder of the mol­ecule and with the dihedral angles of 10.97 (15) and 12.11 (15)°, respectively, between the imidazole ring and the phenyl and methyl­benzene rings; the dihedral angle between the aromatic rings is 8.17 (14)°. Within the urea unit, the N—H atoms are *anti* to each other and one of the N—H atoms forms an intra­molecular N—H⋯N hydrogen bond. Helical supra­molecular chains along [001] are formed *via* N—H⋯N(imidazole) hydrogen bonds in the crystal structure. These are connected into a three-dimensional architecture by C—H⋯O(carbon­yl) and C—H⋯π inter­actions.

## Related literature

For background to epilepsy and epilepsy drugs see: Sander & Shorvon (1987 ▶); Saxena & Saxena (1995 ▶); Edafiogho & Scott (1996 ▶). For the use of aryl semicarbazones as anti-convulsants see: Aboul-Enein *et al.* (2012 ▶); Dimmock *et al.* (1993 ▶, 1995 ▶). For a related structure see: Attia *et al.* (2012 ▶).
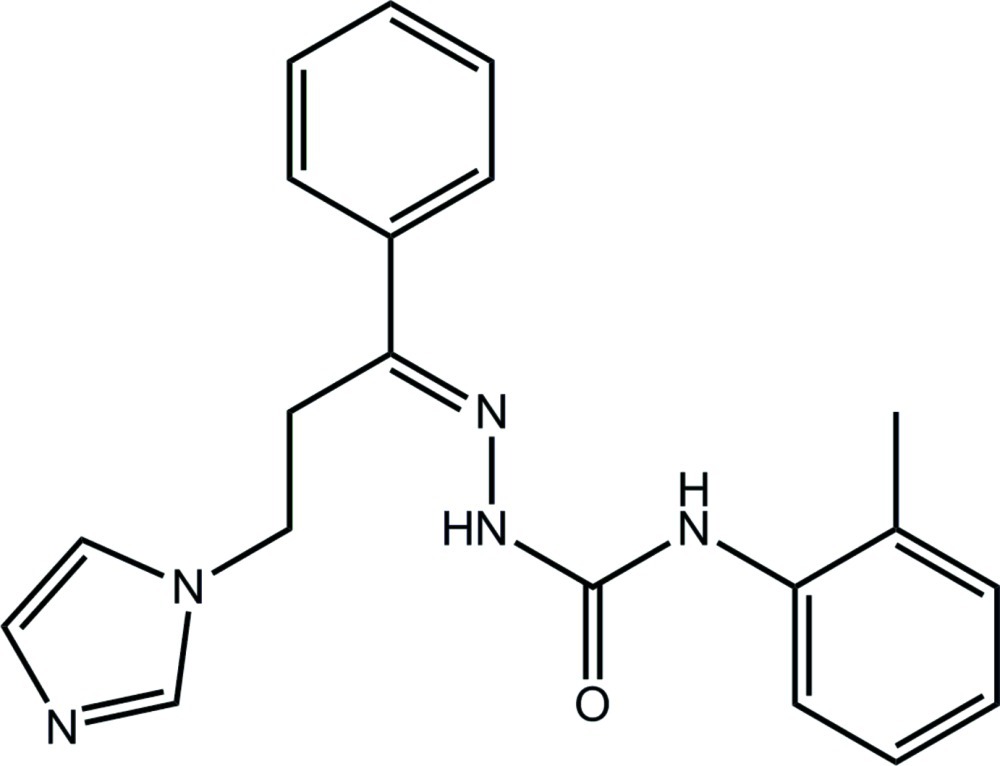


## Experimental

### 

#### Crystal data

C_20_H_21_N_5_O
*M* *_r_* = 347.42
Orthorhombic, 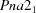

*a* = 20.5220 (17) Å
*b* = 14.1916 (11) Å
*c* = 6.0060 (4) Å
*V* = 1749.2 (2) Å^3^
*Z* = 4
Mo *K*α radiation
μ = 0.09 mm^−1^
*T* = 100 K
0.40 × 0.08 × 0.04 mm

#### Data collection

Agilent SuperNova Dual diffractometer with an Atlas detector
Absorption correction: multi-scan (*CrysAlis PRO*; Agilent, 2011 ▶) *T*_min_ = 0.805, *T*_max_ = 1.000
8422 measured reflections
2211 independent reflections
1809 reflections with *I* > 2σ(*I*)
*R*_int_ = 0.057

#### Refinement

*R*[*F* ^2^ > 2σ(*F* ^2^)] = 0.045
*wR*(*F* ^2^) = 0.097
*S* = 1.02
2211 reflections
244 parameters
3 restraints
H atoms treated by a mixture of independent and constrained refinement
Δρ_max_ = 0.19 e Å^−3^
Δρ_min_ = −0.25 e Å^−3^

### 

Data collection: *CrysAlis PRO* (Agilent, 2011 ▶); cell refinement: *CrysAlis PRO*; data reduction: *CrysAlis PRO*; program(s) used to solve structure: *SHELXS97* (Sheldrick, 2008 ▶); program(s) used to refine structure: *SHELXL97* (Sheldrick, 2008 ▶); molecular graphics: *ORTEP-3* (Farrugia, 1997 ▶) and *DIAMOND* (Brandenburg, 2006 ▶); software used to prepare material for publication: *publCIF* (Westrip, 2010 ▶).

## Supplementary Material

Crystal structure: contains datablock(s) global, I. DOI: 10.1107/S1600536812022659/mw2069sup1.cif

Structure factors: contains datablock(s) I. DOI: 10.1107/S1600536812022659/mw2069Isup2.hkl

Supplementary material file. DOI: 10.1107/S1600536812022659/mw2069Isup3.cml

Additional supplementary materials:  crystallographic information; 3D view; checkCIF report

## Figures and Tables

**Table 1:** Hydrogen-bond geometry (Å, °) *Cg*1 and *Cg*2 are the centroids of the C10–C15 and N4,N5,C18–C20 rings, respectively.

*D*—H⋯*A*	*D*—H	H⋯*A*	*D*⋯*A*	*D*—H⋯*A*
N1—H1*n*⋯N3	0.88 (1)	2.12 (3)	2.601 (3)	114 (2)
N2—H2*n*⋯N5^i^	0.89 (1)	2.03 (1)	2.884 (3)	161 (3)
C5—H5⋯O1^ii^	0.95	2.49	3.416 (3)	164
C7—H7*B*⋯*Cg*1^iii^	0.98	2.82	3.686 (3)	148
C12—H12⋯*Cg*1^iv^	0.95	2.72	3.464 (3)	135
C20—H20⋯*Cg*2^i^	0.95	2.85	3.604 (3)	137

Symmetry codes: (i) 
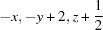
; (ii) 
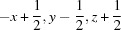
; (iii) 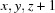; (iv) 
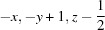
.

**Fig. 1.**
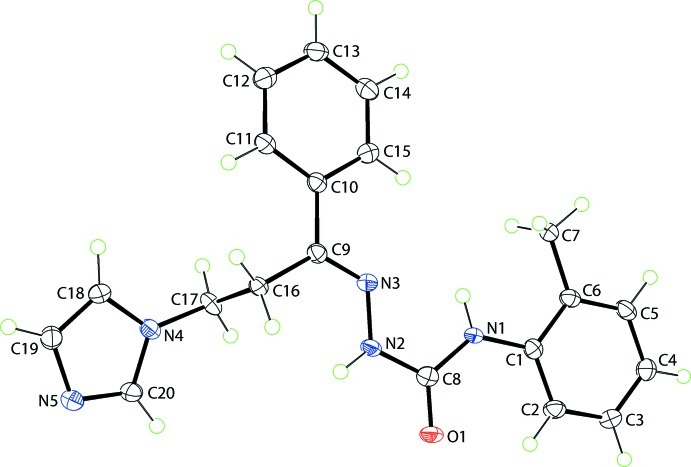
The molecular structure of (I) showing the atom-labelling scheme and displacement ellipsoids at the 50% probability level.

**Fig. 2.**
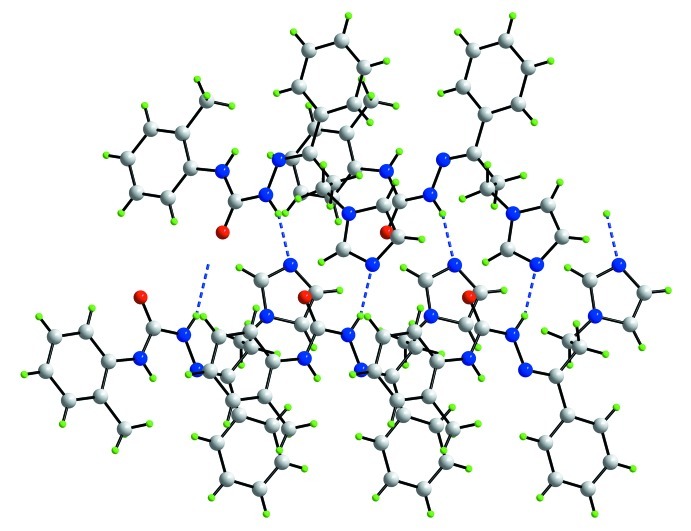
A view of the helical supramolecular chain along [001] in (I) mediated by N—H···N hydrogen bonding, shown as blue dashed lines.

**Fig. 3.**
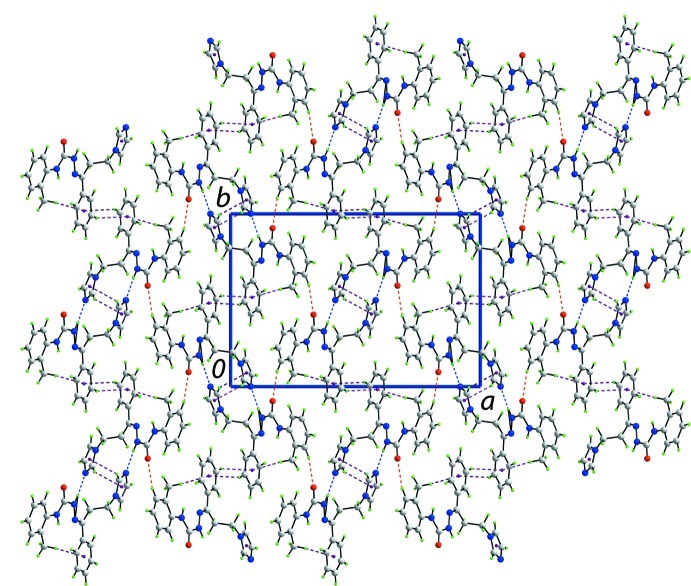
A view in projection down the c axis of the unit-cell contents for (I). The N—H···N, C—H···O and C—H···π interactions are shown as blue, orange and purple dashed lines, respectively.

## supplementary crystallographic information

### Comment

The title compound, (2*E*)-2-[3-(1*H*-imidazol-1-yl)-1-phenylpropylidene]-*N*-(2-methylphenyl)hydrazinecarboxamide (I) will be evaluated as anti-convulsant in experimental animal models for which structural information is desirable. The motivation for its study is the observation that aryl semicarbazones can exhibit significant anti-convulsant activities (Aboul-Enein *et al.*, 2012; Dimmock *et al.*, 1995; Dimmock *et al.*, 1993). Epilepsy is one of the most widespread pathologies of human brain, affecting approximately 1% of world population (Sander & Shorvon, 1987). The need for new drugs arises as currently used anti-epileptic drugs suffer from a number of disadvantages including the fact that approximately one quarter of epileptic patients have seizures that are resistant to the available medical therapies (Saxena & Saxena, 1995). Aside from that, many anti-epileptics used clinically cause significant side-effects (Edafiogho & Scott, 1996).

In (I), Fig. 1, the conformation about the N3═C9 bond [1.289 (3) Å] is *E*. The dihedral angles between the imidazolyl ring and the phenyl and methylbenzene rings are 10.97 (15) and 12.11 (15)°, respectively; the dihedral angle between the phenyl and benzene rings is 8.17 (14)°. Overall, the main part of the molecule, excepting the imidazolyl substituent, appears flat and is significantly flatter than the recently determined 4-methoxybenzene analogue (Attia *et al.*, 2012). Within the urea moiety, the N—H atoms are *anti* to each other and the N1—H atom forms an intramolecular N—H···N hydrogen bond which defines a *S*(5) loop, Table 1.

In the crystal structure, helical supramolecular chains along [001] are formed *via* N—H···N(imidazolyl) hydrogen bonds, Fig. 2 and Table 1. These are connected into a three-dimensional architecture by *C*—*H*···*O*(carbonyl) and C—H···π interactions, Fig. 3 and Table 1.

### Experimental

Acetic acid (2 drops) was added to a stirred solution of 3-(1*H*-imidazol-1-yl)-1-phenyl-propan-1-one (0.20 g, 1 mmol) and *N*-(2-methylphenyl)hydrazinecarboxamide (0.17 g, 1 mmol) in absolute ethanol (10 mL). The reaction mixture was stirred at room temperature for 18 h. The solution was concentrated under vacuum and the precipitated solid was filtered off. The collected solid was recrystallized from ethanol to give crystals of the title compound; *M.P.*: 453–455 K.

### Refinement

Carbon-bound H-atoms were placed in calculated positions [C—H = 0.95 to 0.99 Å, *U*_iso_(H) = 1.2–1.5*U*_eq_(C)] and were included in the refinement in the riding model approximation. The amino H-atoms were refined with N—H = 0.88±0.01 Å. In the absence of significant anomalous scattering effects, 1613 Friedel pairs were averaged in the final refinement.

### Crystal data

**Table:**

C_20_H_21_N_5_O	*F*(000) = 736
*M**_r_* = 347.42	*D*_x_ = 1.319 Mg m^−^^3^
Orthorhombic, *P**n**a*2_1_	Mo *K*α radiation, λ = 0.71073 Å
Hall symbol: P 2c -2n	Cell parameters from 2304 reflections
*a* = 20.5220 (17) Å	θ = 2.5–27.5°
*b* = 14.1916 (11) Å	µ = 0.09 mm^−^^1^
*c* = 6.0060 (4) Å	*T* = 100 K
*V* = 1749.2 (2) Å^3^	Prism, colourless
*Z* = 4	0.40 × 0.08 × 0.04 mm

### Data collection

**Table:**

Agilent SuperNova Dual diffractometer with an Atlas detector	2211 independent reflections
Radiation source: SuperNova (Mo) X-ray Source	1809 reflections with *I* > 2σ(*I*)
Mirror monochromator	*R*_int_ = 0.057
Detector resolution: 10.4041 pixels mm^-1^	θ_max_ = 27.6°, θ_min_ = 2.5°
ω scan	*h* = −26→25
Absorption correction: multi-scan (*CrysAlis PRO*; Agilent, 2011)	*k* = −18→14
*T*_min_ = 0.805, *T*_max_ = 1.000	*l* = −7→7
8422 measured reflections	

### Refinement

**Table:**

Refinement on *F*^2^	Primary atom site location: structure-invariant direct methods
Least-squares matrix: full	Secondary atom site location: difference Fourier map
*R*[*F*^2^ > 2σ(*F*^2^)] = 0.045	Hydrogen site location: inferred from neighbouring sites
*wR*(*F*^2^) = 0.097	H atoms treated by a mixture of independent and constrained refinement
*S* = 1.02	*w* = 1/[σ^2^(*F*_o_^2^) + (0.0372*P*)^2^ + 0.4249*P*] where *P* = (*F*_o_^2^ + 2*F*_c_^2^)/3
2211 reflections	(Δ/σ)_max_ < 0.001
244 parameters	Δρ_max_ = 0.19 e Å^−^^3^
3 restraints	Δρ_min_ = −0.25 e Å^−^^3^

### Special details

**Table:**

Geometry. All e.s.d.'s (except the e.s.d. in the dihedral angle between two l.s. planes) are estimated using the full covariance matrix. The cell e.s.d.'s are taken into account individually in the estimation of e.s.d.'s in distances, angles and torsion angles; correlations between e.s.d.'s in cell parameters are only used when they are defined by crystal symmetry. An approximate (isotropic) treatment of cell e.s.d.'s is used for estimating e.s.d.'s involving l.s. planes.
Refinement. Refinement of *F*^2^ against ALL reflections. The weighted *R*-factor *wR* and goodness of fit *S* are based on *F*^2^, conventional *R*-factors *R* are based on *F*, with *F* set to zero for negative *F*^2^. The threshold expression of *F*^2^ > σ(*F*^2^) is used only for calculating *R*-factors(gt) *etc*. and is not relevant to the choice of reflections for refinement. *R*-factors based on *F*^2^ are statistically about twice as large as those based on *F*, and *R*- factors based on ALL data will be even larger.

### Fractional atomic coordinates and isotropic or equivalent isotropic displacement parameters (Å^2^)

**Table:**

	*x*	*y*	*z*	*U*_iso_*/*U*_eq_	
O1	0.16812 (10)	0.91634 (13)	1.0006 (3)	0.0235 (5)	
N1	0.19630 (12)	0.75898 (15)	1.0029 (4)	0.0190 (5)	
H1n	0.1888 (15)	0.7078 (14)	0.925 (5)	0.029 (9)*	
N2	0.12686 (12)	0.81951 (15)	0.7377 (4)	0.0198 (5)	
H2n	0.1117 (13)	0.8684 (14)	0.662 (4)	0.016 (8)*	
N3	0.12298 (12)	0.72925 (16)	0.6568 (4)	0.0180 (5)	
N4	−0.04027 (11)	0.86786 (16)	0.2322 (4)	0.0196 (5)	
N5	−0.08081 (12)	0.99922 (16)	0.0881 (4)	0.0213 (5)	
C1	0.23540 (14)	0.7509 (2)	1.1950 (4)	0.0179 (6)	
C2	0.24925 (15)	0.8274 (2)	1.3333 (5)	0.0214 (6)	
H2	0.2335	0.8884	1.2963	0.026*	
C3	0.28578 (14)	0.8145 (2)	1.5240 (5)	0.0241 (7)	
H3	0.2945	0.8666	1.6188	0.029*	
C4	0.30981 (16)	0.7261 (2)	1.5779 (5)	0.0241 (7)	
H4	0.3350	0.7174	1.7090	0.029*	
C5	0.29672 (14)	0.65011 (19)	1.4380 (5)	0.0218 (6)	
H5	0.3132	0.5896	1.4753	0.026*	
C6	0.26012 (13)	0.66097 (19)	1.2451 (5)	0.0185 (6)	
C7	0.24667 (14)	0.57690 (18)	1.0977 (5)	0.0209 (6)	
H7A	0.2613	0.5905	0.9458	0.031*	
H7B	0.1998	0.5638	1.0967	0.031*	
H7C	0.2702	0.5219	1.1550	0.031*	
C8	0.16516 (13)	0.83647 (19)	0.9221 (4)	0.0186 (6)	
C9	0.09415 (13)	0.71323 (19)	0.4699 (5)	0.0177 (6)	
C10	0.09067 (13)	0.61251 (19)	0.4007 (5)	0.0171 (6)	
C11	0.06162 (14)	0.58577 (19)	0.1998 (5)	0.0196 (6)	
H11	0.0447	0.6328	0.1033	0.023*	
C12	0.05714 (14)	0.4915 (2)	0.1391 (5)	0.0230 (7)	
H12	0.0368	0.4746	0.0026	0.028*	
C13	0.08204 (15)	0.4225 (2)	0.2759 (5)	0.0242 (7)	
H13	0.0787	0.3581	0.2344	0.029*	
C14	0.11191 (14)	0.4473 (2)	0.4744 (5)	0.0251 (7)	
H14	0.1293	0.3998	0.5687	0.030*	
C15	0.11650 (15)	0.54123 (19)	0.5355 (5)	0.0226 (6)	
H15	0.1375	0.5575	0.6711	0.027*	
C16	0.06317 (14)	0.78834 (19)	0.3273 (4)	0.0181 (6)	
H16A	0.0663	0.7698	0.1688	0.022*	
H16B	0.0872	0.8482	0.3465	0.022*	
C17	−0.00841 (14)	0.80344 (19)	0.3886 (5)	0.0217 (6)	
H17A	−0.0314	0.7421	0.3874	0.026*	
H17B	−0.0113	0.8297	0.5410	0.026*	
C18	−0.06042 (14)	0.8465 (2)	0.0194 (5)	0.0220 (6)	
H18	−0.0575	0.7871	−0.0529	0.026*	
C19	−0.08538 (14)	0.92777 (19)	−0.0665 (5)	0.0220 (6)	
H19	−0.1033	0.9342	−0.2116	0.026*	
C20	−0.05398 (14)	0.96020 (19)	0.2651 (5)	0.0206 (6)	
H20	−0.0453	0.9930	0.3998	0.025*	

### Atomic displacement parameters (Å^2^)

**Table:**

	*U*^11^	*U*^22^	*U*^33^	*U*^12^	*U*^13^	*U*^23^
O1	0.0321 (11)	0.0115 (9)	0.0270 (11)	0.0001 (9)	−0.0050 (10)	−0.0016 (8)
N1	0.0223 (13)	0.0119 (11)	0.0227 (12)	0.0029 (10)	−0.0052 (11)	−0.0031 (10)
N2	0.0275 (13)	0.0124 (11)	0.0196 (12)	0.0038 (10)	−0.0040 (11)	−0.0005 (10)
N3	0.0197 (12)	0.0143 (12)	0.0200 (12)	−0.0002 (10)	−0.0003 (10)	−0.0008 (8)
N4	0.0201 (12)	0.0165 (12)	0.0222 (12)	0.0026 (10)	−0.0004 (11)	0.0012 (10)
N5	0.0230 (13)	0.0174 (12)	0.0235 (12)	0.0022 (11)	0.0003 (12)	−0.0003 (10)
C1	0.0150 (13)	0.0194 (15)	0.0192 (14)	0.0002 (11)	0.0023 (12)	0.0009 (11)
C2	0.0225 (15)	0.0148 (13)	0.0268 (15)	−0.0033 (12)	0.0011 (12)	0.0001 (12)
C3	0.0253 (15)	0.0199 (15)	0.0272 (16)	−0.0016 (13)	−0.0037 (14)	−0.0054 (12)
C4	0.0267 (16)	0.0246 (15)	0.0210 (14)	0.0014 (13)	−0.0065 (13)	0.0010 (12)
C5	0.0213 (14)	0.0156 (14)	0.0284 (16)	0.0030 (12)	0.0008 (14)	0.0039 (12)
C6	0.0180 (14)	0.0150 (14)	0.0226 (14)	−0.0004 (11)	0.0032 (14)	−0.0004 (12)
C7	0.0199 (15)	0.0162 (14)	0.0265 (14)	0.0023 (12)	−0.0038 (13)	−0.0009 (12)
C8	0.0184 (14)	0.0172 (13)	0.0202 (14)	−0.0022 (12)	0.0040 (13)	0.0019 (11)
C9	0.0164 (14)	0.0160 (14)	0.0206 (14)	−0.0003 (11)	0.0006 (12)	0.0015 (11)
C10	0.0145 (13)	0.0170 (13)	0.0199 (13)	0.0003 (11)	0.0017 (12)	0.0007 (11)
C11	0.0198 (14)	0.0169 (14)	0.0221 (14)	0.0016 (12)	0.0005 (13)	0.0017 (11)
C12	0.0189 (15)	0.0250 (16)	0.0252 (16)	−0.0017 (13)	0.0026 (13)	−0.0048 (12)
C13	0.0244 (16)	0.0173 (14)	0.0309 (16)	0.0004 (13)	0.0013 (15)	−0.0048 (12)
C14	0.0265 (16)	0.0205 (15)	0.0283 (16)	0.0046 (13)	−0.0004 (14)	0.0013 (12)
C15	0.0248 (16)	0.0195 (14)	0.0235 (15)	−0.0007 (13)	−0.0029 (13)	−0.0021 (12)
C16	0.0223 (15)	0.0138 (13)	0.0181 (13)	0.0023 (12)	−0.0034 (12)	0.0020 (10)
C17	0.0231 (16)	0.0185 (14)	0.0234 (15)	0.0061 (12)	0.0013 (13)	0.0060 (12)
C18	0.0262 (15)	0.0197 (14)	0.0200 (14)	0.0020 (13)	−0.0024 (14)	−0.0048 (11)
C19	0.0216 (15)	0.0224 (15)	0.0220 (15)	0.0001 (12)	−0.0021 (13)	0.0012 (12)
C20	0.0216 (14)	0.0178 (14)	0.0223 (14)	0.0022 (12)	0.0007 (13)	−0.0032 (11)

### Geometric parameters (Å, º)

**Table:**

O1—C8	1.229 (3)	C7—H7B	0.9800
N1—C8	1.361 (3)	C7—H7C	0.9800
N1—C1	1.410 (4)	C9—C10	1.490 (4)
N1—H1n	0.879 (10)	C9—C16	1.508 (4)
N2—N3	1.373 (3)	C10—C11	1.398 (4)
N2—C8	1.379 (4)	C10—C15	1.400 (4)
N2—H2n	0.885 (10)	C11—C12	1.390 (4)
N3—C9	1.289 (3)	C11—H11	0.9500
N4—C20	1.355 (3)	C12—C13	1.377 (4)
N4—C18	1.377 (4)	C12—H12	0.9500
N4—C17	1.465 (3)	C13—C14	1.386 (4)
N5—C20	1.319 (4)	C13—H13	0.9500
N5—C19	1.378 (4)	C14—C15	1.386 (4)
C1—C2	1.396 (4)	C14—H14	0.9500
C1—C6	1.406 (4)	C15—H15	0.9500
C2—C3	1.381 (4)	C16—C17	1.530 (4)
C2—H2	0.9500	C16—H16A	0.9900
C3—C4	1.387 (4)	C16—H16B	0.9900
C3—H3	0.9500	C17—H17A	0.9900
C4—C5	1.393 (4)	C17—H17B	0.9900
C4—H4	0.9500	C18—C19	1.363 (4)
C5—C6	1.389 (4)	C18—H18	0.9500
C5—H5	0.9500	C19—H19	0.9500
C6—C7	1.511 (4)	C20—H20	0.9500
C7—H7A	0.9800		
			
C8—N1—C1	128.6 (2)	C10—C9—C16	120.0 (2)
C8—N1—H1n	113 (2)	C11—C10—C15	117.7 (2)
C1—N1—H1n	118 (2)	C11—C10—C9	121.4 (2)
N3—N2—C8	118.7 (2)	C15—C10—C9	120.9 (2)
N3—N2—H2n	121.9 (19)	C12—C11—C10	121.1 (3)
C8—N2—H2n	118.4 (19)	C12—C11—H11	119.5
C9—N3—N2	120.0 (2)	C10—C11—H11	119.5
C20—N4—C18	106.6 (2)	C13—C12—C11	120.2 (3)
C20—N4—C17	127.1 (2)	C13—C12—H12	119.9
C18—N4—C17	126.3 (2)	C11—C12—H12	119.9
C20—N5—C19	105.2 (2)	C12—C13—C14	119.8 (3)
C2—C1—C6	120.3 (3)	C12—C13—H13	120.1
C2—C1—N1	122.7 (3)	C14—C13—H13	120.1
C6—C1—N1	117.0 (2)	C13—C14—C15	120.1 (3)
C3—C2—C1	120.1 (3)	C13—C14—H14	119.9
C3—C2—H2	120.0	C15—C14—H14	119.9
C1—C2—H2	120.0	C14—C15—C10	121.1 (3)
C2—C3—C4	120.4 (3)	C14—C15—H15	119.5
C2—C3—H3	119.8	C10—C15—H15	119.5
C4—C3—H3	119.8	C9—C16—C17	111.6 (2)
C3—C4—C5	119.4 (3)	C9—C16—H16A	109.3
C3—C4—H4	120.3	C17—C16—H16A	109.3
C5—C4—H4	120.3	C9—C16—H16B	109.3
C6—C5—C4	121.4 (3)	C17—C16—H16B	109.3
C6—C5—H5	119.3	H16A—C16—H16B	108.0
C4—C5—H5	119.3	N4—C17—C16	111.2 (2)
C5—C6—C1	118.3 (3)	N4—C17—H17A	109.4
C5—C6—C7	120.0 (2)	C16—C17—H17A	109.4
C1—C6—C7	121.7 (3)	N4—C17—H17B	109.4
C6—C7—H7A	109.5	C16—C17—H17B	109.4
C6—C7—H7B	109.5	H17A—C17—H17B	108.0
H7A—C7—H7B	109.5	C19—C18—N4	106.1 (3)
C6—C7—H7C	109.5	C19—C18—H18	126.9
H7A—C7—H7C	109.5	N4—C18—H18	126.9
H7B—C7—H7C	109.5	C18—C19—N5	110.0 (3)
O1—C8—N1	125.8 (3)	C18—C19—H19	125.0
O1—C8—N2	119.8 (2)	N5—C19—H19	125.0
N1—C8—N2	114.4 (2)	N5—C20—N4	112.0 (3)
N3—C9—C10	115.7 (2)	N5—C20—H20	124.0
N3—C9—C16	124.3 (2)	N4—C20—H20	124.0
			
C8—N2—N3—C9	−171.6 (2)	N3—C9—C10—C15	1.1 (4)
C8—N1—C1—C2	−1.6 (5)	C16—C9—C10—C15	−177.1 (3)
C8—N1—C1—C6	177.8 (3)	C15—C10—C11—C12	1.6 (4)
C6—C1—C2—C3	−1.9 (4)	C9—C10—C11—C12	−178.4 (3)
N1—C1—C2—C3	177.5 (3)	C10—C11—C12—C13	−0.6 (4)
C1—C2—C3—C4	1.0 (5)	C11—C12—C13—C14	−0.3 (4)
C2—C3—C4—C5	−0.1 (5)	C12—C13—C14—C15	0.3 (4)
C3—C4—C5—C6	0.1 (5)	C13—C14—C15—C10	0.7 (4)
C4—C5—C6—C1	−1.0 (4)	C11—C10—C15—C14	−1.6 (4)
C4—C5—C6—C7	−179.8 (3)	C9—C10—C15—C14	178.4 (3)
C2—C1—C6—C5	1.9 (4)	N3—C9—C16—C17	−90.0 (3)
N1—C1—C6—C5	−177.6 (3)	C10—C9—C16—C17	88.0 (3)
C2—C1—C6—C7	−179.3 (3)	C20—N4—C17—C16	−101.8 (3)
N1—C1—C6—C7	1.2 (4)	C18—N4—C17—C16	76.1 (3)
C1—N1—C8—O1	3.3 (5)	C9—C16—C17—N4	−173.0 (2)
C1—N1—C8—N2	−175.7 (3)	C20—N4—C18—C19	0.1 (3)
N3—N2—C8—O1	−178.3 (2)	C17—N4—C18—C19	−178.1 (3)
N3—N2—C8—N1	0.8 (4)	N4—C18—C19—N5	0.3 (3)
N2—N3—C9—C10	−178.0 (2)	C20—N5—C19—C18	−0.7 (3)
N2—N3—C9—C16	0.0 (4)	C19—N5—C20—N4	0.8 (3)
N3—C9—C10—C11	−179.0 (3)	C18—N4—C20—N5	−0.6 (3)
C16—C9—C10—C11	2.9 (4)	C17—N4—C20—N5	177.7 (3)

### Hydrogen-bond geometry (Å, º)

Cg1 and Cg2 are the centroids of the C10–C15 and N4,N5,C18–C20 rings, respectively.

**Table:**

*D*—H···*A*	*D*—H	H···*A*	*D*···*A*	*D*—H···*A*
N1—H1*n*···N3	0.88 (1)	2.12 (3)	2.601 (3)	114 (2)
N2—H2*n*···N5^i^	0.89 (1)	2.03 (1)	2.884 (3)	161 (3)
C5—H5···O1^ii^	0.95	2.49	3.416 (3)	164
C7—H7*B*···*Cg*1^iii^	0.98	2.82	3.686 (3)	148
C12—H12···*Cg*1^iv^	0.95	2.72	3.464 (3)	135
C20—H20···*Cg*2^i^	0.95	2.85	3.604 (3)	137

Symmetry codes: (i) −*x*, −*y*+2, *z*+1/2; (ii) −*x*+1/2, *y*−1/2, *z*+1/2; (iii) *x*, *y*, *z*+1; (iv) −*x*, −*y*+1, *z*−1/2.
